# Differential optogenetic activation of the auditory midbrain in freely moving behaving mice

**DOI:** 10.3389/fnsys.2023.1222176

**Published:** 2023-08-31

**Authors:** Meike M. Rogalla, Adina Seibert, Jana M. Sleeboom, K. Jannis Hildebrandt

**Affiliations:** ^1^Department of Neuroscience, Division of Auditory Neuroscience, Carl von Ossietzky University, Oldenburg, Lower Saxony, Germany; ^2^Cluster of Excellence Hearing4all, Carl von Ossietzky University, Oldenburg, Lower Saxony, Germany

**Keywords:** auditory midbrain implant, optogenetic stimulation, inferior colliculus, Go/No-Go, auditory prosthesis

## Abstract

**Introduction:**

In patients with severe auditory impairment, partial hearing restoration can be achieved by sensory prostheses for the electrical stimulation of the central nervous system. However, these state-of-the-art approaches suffer from limited spectral resolution: electrical field spread depends on the impedance of the surrounding medium, impeding spatially focused electrical stimulation in neural tissue. To overcome these limitations, optogenetic activation could be applied in such prostheses to achieve enhanced resolution through precise and differential stimulation of nearby neuronal ensembles. Previous experiments have provided a first proof for behavioral detectability of optogenetic activation in the rodent auditory system, but little is known about the generation of complex and behaviorally relevant sensory patterns involving differential activation.

**Methods:**

In this study, we developed and behaviorally tested an optogenetic implant to excite two spatially separated points along the tonotopy of the murine inferior colliculus (ICc).

**Results:**

Using a reward based operant Go/No-Go paradigm, we show that differential optogenetic activation of a sub-cortical sensory pathway is possible and efficient. We demonstrate how animals which were previously trained in a frequency discrimination paradigm (a) rapidly respond to either sound or optogenetic stimulation, (b) generally detect optogenetic stimulation of two different neuronal ensembles, and (c) discriminate between them.

**Discussion:**

Our results demonstrate that optogenetic excitatory stimulation at different points of the ICc tonotopy elicits a stable response behavior over time periods of several months. With this study, we provide the first proof of principle for sub-cortical differential stimulation of sensory systems using complex artificial cues in freely moving animals.

## Introduction

Implants restoring auditory function in deaf patients represent the most successful neuronal prostheses. Cochlear implants (CI) alone enabled hearing in over 1 million patients worldwide (Zeng, 2022). However, in the presence of pathological conditions where CI cannot be implanted [e.g., aplasia, ossification of the cochlea, or neurofibromatosis type 2 (NF2)-derived bilateral acoustic neuromas], partial restoration of hearing can only be achieved via central sensory prostheses (Middlebrooks et al., 2005; Zeng et al., 2008; Moore and Shannon, 2009; NIDCD, 2015). Thus, electrical approaches have been developed to stimulate central circuits of the auditory pathway to restore auditory perception in humans (Colletti et al., 2009; Lim and Lenarz, 2015). One solution is the electrical stimulation of the cochlear nucleus (CN) via an auditory brainstem implant (ABI), which consists of either an electrode surface array (e.g., McInturff et al., 2023) or penetrating array (PABI, e.g., McCreery et al., 2018). Although the PABI provides lower thresholds and a greater selectivity (Shannon, 2012), no difference in speech understanding could be observed between patients with PABIs or surface arrays (Otto et al., 2008; Wong et al., 2019). However, implantation outcome in general varies strongly among individuals and many recipients reach a much lower performance than CI patients, especially in speech perception (Colletti and Shannon, 2005; Lim et al., 2008, 2009; Colletti et al., 2009). This drastic difference is suggested to be caused by tumor-related tissue damage or abnormal anatomy in NF2 patients (Otto et al., 2002; Lim et al., 2009; Matthies et al., 2014; Wong et al., 2019).

To overcome these limitations for NF2 patients, implants have been developed to stimulate the central inferior colliculus (ICc) (Lenarz et al., 2006; Lim et al., 2009). Electrical stimulation of the ICc represents a promising target for specific stimulation due to its well-defined frequency-laminae and tonotopic organization, possibly resulting in high frequency resolution (Colletti et al., 2007; Lim et al., 2007, 2009; Lim and Lenarz, 2015). Electrical stimulation of the central nervous system has, however, limited temporal and, especially, spatial resolution, since electrical field spread impedes spatially focused stimulation, resulting in a wide spread of excitation across tonotopic laminae (Kral et al., 1998; Hernandez et al., 2014). Results of the first clinical trial of electrical stimulation of the human IC were modest and complex stimulus perception could not be achieved (Lim et al., 2007; Lim and Lenarz, 2015).

Thus, alternative approaches are required to achieve differential stimulation of neuronal ensembles of the auditory pathway, especially when those are located in the central nervous system. Optogenetic stimulation via genetically encoded light-activated ion channels could be applied to neuronal prostheses to achieve more precise excitation (Bernstein and Boyden, 2011; Fenno et al., 2011; Mattis et al., 2012; Delbeke et al., 2017), resulting in enhanced resolution (Hernandez et al., 2014; Moser, 2015; Moser and Dieter, 2020). In theory, such differential stimulation should enable the artificial generation of complex auditory percepts.

The behavioral detectability of optogenetic activation has successfully been demonstrated in the rodent auditory pathway (Guo et al., 2015; Wrobel et al., 2018), providing the first proof-of principle for its feasibility of generating behaviorally relevant percepts. Additionally, optogenetic stimulation of superficial cortical neurons has already been shown to “mimic” complex sensory percepts (Ceballo et al., 2019), paving the way for the feasibility of optogenetics in prostheses. Particularly this superficial optogenetic activation of the auditory cortex will be difficult for implementing prostheses-generated behaviorally relevant sensations in patients. However, penetrating, differential stimulation of the sub-cortical sensory nuclei like the IC may be a promising approach to generate artificial sensory cues and could eventually replace electrical implants in the future.

In this study we devised and tested a novel unilateral optogenetic auditory midbrain implant (oAMI) for the activation of two spatially separated points along the tonotopy of the murine central ICc. A reward-based operant Go/No-Go paradigm permitted evaluating optogenetic activation of the auditory midbrain compared to sound stimulation in freely moving, behaving mice. We demonstrate how the resulting percept reliably drives behavior: mice previously trained in a sound frequency discrimination task (1) rapidly generalized between acoustical and artificial cues, (2) generally detected optogenetic stimulation at two separated points of the ICc tonotopy, and (3) discriminated between them. Optogenetic activation at different points of the ICc tonotopy was reliably detected and discriminated over several months. We thus not only uniquely provide a flexible reward-based paradigm for the controlled behavioral evaluation of optogenetic activation of the rodent auditory pathway, but also the first proof-of-principle for sub-cortical differential stimulation of sensory systems using complex artificial cues in freely moving animals.

## Materials and methods

### Animals

All experiments were performed in accordance with the animal welfare regulations of Lower Saxony and with the approval from the local authorities (State Office for Consumer Protection and Food Safety/LAVES, permission number 33.9-43502-04-18/2802).

Mice had a C57BL/6.CAST-Cdh23*^Ahl^*^+^ background (the Jackson Laboratory, #002756). Standardized cages were used for housing, equipped with cage enrichment. Animals were single housed but with visual and olfactory contact to neighboring animals at a reversed 12/12 h dark-light cycle. Experiments were conducted during the dark period for 1–2 times/day. During experimental periods, animals had unlimited access to water but were food-deprived to a moderate extent (85–90% of their ad libitum weight). Weight and wellbeing were scored daily.

In total, 10 adult male mice, bred at the University of Oldenburg animal facilities, were used, 8 animals in the test group and 2 mice served as control animals. Each experiment has only been performed by a subset of mice. For more information, see Supplementary Table 1.

### Implants and surgery

#### Implants

**FIGURE 1:**
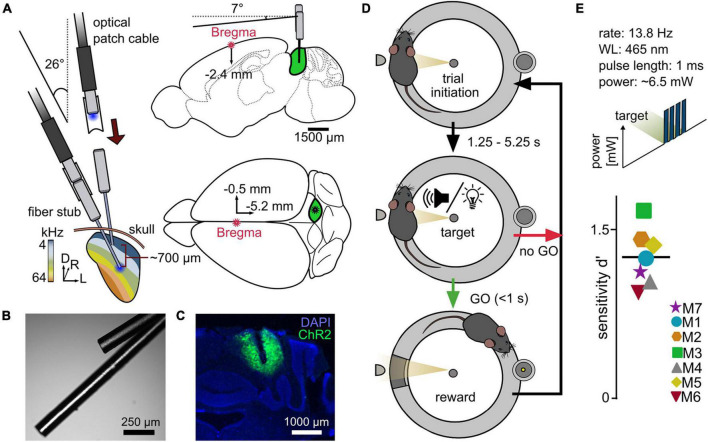
A reward-based operant Go/No-Go paradigm for the evaluation of differential optogenetic activation of the ICc. **(A)** Simplified implantation strategy. Animals underwent a craniotomy followed by injection of the viral construct (rAAV-CAG-ChR2-GFP, 0.9 μl) into the right central inferior colliculus (ICc), followed by an implantation of two optical outlets. Optical fibers for LED stimulation with a diameter of 110 μm were used. **(B)** Example image of optical outlets. Note that it was ensured during the assembly that optical fibers did not point toward each other to avoid incoupling. **(C)** Viral expression within the IC + DAPI (blue). **(D)** Go/No-Go paradigm. The setup consisted of a circular runway, lined with plexiglass walls and equipped with a platform. The mouse initiated a trial via staying on the platform. After a random delay, the stimulus was presented. If the mouse indicated stimulus recognition by leaving the platform within a time window of one second, a pellet was released as reward at the opposite side of the setup. **(E)** Proof of principle – optogenetic activation of the ICc. Mice performed a paradigm for the detection of optogenetic light pulse trains with a constant total power of ∼6.5 mW at either one of the two stimulation points. The overall sensitivity for each animal is shown; black line gives the mean over seven animals.

The oAMI consisted of two separate fiber outlets with a difference in depth of 700 μm, terminating at two different positions with the right IC (dorsal and ventral, see Figures 1A–C). PlexBright fiber stub implants (Plexon Inc.) were used with LC ceramic ferrules (1.25 mm outer diameter, 6.45 mm length), equipped with 1.2 cm long high-performance optical fibers of a diameter of 110/125 μm and a numerical aperture (NA) of 0.66. At the distal region of the ferrule, a small notch was cut with a diamond plate grinder for a better grip of the dental cement (Vertex Self-Curing, Vertex Dental). Prior to attachment, fibers and ferrules were cleaned with dust-free wipes and isopropanol. Fiber stubs were aligned with a distance of 700 μm under a microscope and covered with dental cement, from the medial part of the fibers leaving ∼1/3 exposed, to the medial part of the ferrules. Both outlets were tested for their functionality and stored in dust-free plastic boxes until implantation. Levels of light output in each implant at corresponding voltage values were verified at the fiber stub tips using a digital optical power and energy meter (PM100D, Thorlabs) in combination with a photodiode power sensor (S121C, Thorlabs) and an oscilloscope to ensure that both outlets had identical emission profiles.

#### Histology

Implant position (when the animal has not lost the implant during experiments) and virus expression were evaluated in frozen sections (40 μm) of perfused and coronal sliced brains (example in Figure 1C). Slices were additionally stained with DAPI (1 μg/ml, D9542, Lot #096M4014V, Sigma).

#### Transfection and implantation

Mice had unlimited access to food for at least 3 days prior to surgery. Anesthesia was induced using 2% isoflurane (CP Pharma) provided with oxygen and a flowrate of approximately 0.9 l/min. Animals received 0.1 mg/kg meloxicam (Metacam, 2 mg/ml, Boehringer Ingelheim) in Ringer-lactate solution subcutaneously. Surgery was performed on a heating pad in a stereotactic frame for rodents (Model 900, Kopf Instruments) using zygomatic bars. The head was fixated with an angle of 7° in the rostrocaudal axis to reach the same *z*-position for Bregma and IC (see Figure 1A). After proper fixation, anesthesia was reduced to the maintenance dose of 1.2–1.5%. A small incision was made from the caudal end of the frontal bone near the coronal suture and extended caudally until full exposure of the interparietal bone. The periost was removed and 4–5 holes were drilled into the parietal bones and equipped with autoclaved screws. Bregma and Lambda were determined, and a small trepanation was drilled above the right IC (*X* = 0.5; *Y* = −5.2, mm relative to Bregma). The viral construct (rAAV5-CAG-ChR2-GFP, 2.9 × 10^12^ molecules/ml, Lot# AV4597D, UNC GTC Vector Core) was injected using a 10 μl syringe and an ultra-micro pump (UMP3 with SYS-Micro4 Controller, WPI) and 35 G needles on a micron-resolution manipulator. 3 × 300 nl of the construct were injected at an angle of −26° relative to Bregma (*Z*^1^ = −1.5, *Z*^2^ = −2.1, *Z*^3^ = −2.7 mm) with a rate of 150 nl/min. After each injection, the needle was maintained at its position for 5 min. Implantation was performed using a custom-made tool adjusted on a standard cannula holder on the manipulator using the same *X* and *Y* coordinates as for the injection, with the ventral outlet as reference. The oAMI was implanted at *Z* = −2.4 mm (Figures 1A–C), the craniotomy was covered with sterile Vaseline and the implant and screws were completely covered with dental cement. A second dose of meloxicam in Ringer solution was administered subcutaneously. Throughout the surgery and a postsurgical period of 2–3 h, breathing frequency and wellbeing were monitored and the temperature was kept at ∼37°C.

During a recovery period of 2 weeks, animals received 1.77 mg/kg meloxicam (Metacam, 0.5 mg/ml, Boehringer Ingelheim, Germany) orally, mixed with a high-energy nutritional supplement (DietGel Boost, ClearH_2_O) every 12 h for at least 7 days. During the whole period, animals had unlimited access to water and food.

### Behavioral experiments

#### Behavioral setup and Go/No-Go paradigm

Animals were placed on an elevated circular shaped wire mesh runway, lined with outer and inner walls of Plexiglas (outer Ø = 29 cm, inner Ø = 20 cm, height = 27 cm, Figure 1D) positioned in a double-walled sound attenuated booth with pyramid foam covered walls (Industrial Acoustics Company GmbH). One side of the runway was equipped with a small platform (5 × 3 × 4 cm). Once the animals ascended the platform, which was detected by a light barrier, a random waiting time started, ranging from 1.25 to 5.25 s in steps of 1 s, followed by a target presentation. The onset of the target triggered a 1 s response window. If the animals descended from the platform within the window (“go”), a food pellet (0.02 g, Dustless precision pellets rodent, grain based, #F0163, Bio-Serv) was delivered at the opposite side of the runway by a feeder (hit). If the animals did not leave the platform (miss), a new trial was presented after a newly drawn waiting time. To estimate the amount of coincidently correct responded trials, 27.27% of trials were sham trials (trials without stimulus presentation), pseudo-randomly integrated into each session (Figure 1D). These trials had the same distribution of waiting times as the target trials and neither a response to (false alarm), nor a correct rejection of the sham trials were punished or rewarded. A session contained 40 trials and 15 sham trials and usually lasted 15–30 min. Sessions were usually performed twice/day.

To reduce the possibility of visual detection of the light stimulation, the booth was additionally equipped with a blue light LED strip at the ceiling (57 cm, 30 LEDs). This masking LED was continuously pulsing in the same rate as the chosen stimulation task during every single session, also when using sound only.

Behavioral experiments were controlled by a custom Software (PsychDetect, Github repository^1^), written in MATLAB (The Mathworks, RRID:SCR_001622). Pellet dispenser and light barriers were custom made (University of Oldenburg workshop) and controlled by a microcontroller (Arduino UNO, Arduino AG) connected to a Windows PC. Experiments were carried out in darkness and were observed visually by a camera with infrared LEDs, running on a raspberry Pi (Model 3B+).

#### Sound stimulation

A speaker (Vifa XT 300/K4) was placed at the ceiling of the booth, approximately 0.8 m above the platform. Sound was generated using a high-fidelity sound card (Fireface UC, RME) connected to the PC. Sound was played back at 96 kHz sampling rate. The speaker was calibrated on the platform at the approximate position of the head of the animals using a measurement microphone (model 40BF, G.R.A.S). Calibration was done using tone pips of 10 ms duration, played with a resolution of 24 tones per octave. The sound system frequency response was flat within ±1.5 dB across the calibrated range.

##### Handling and auditory training

Upon arrival from the local animal facility at the age of 9–12 weeks, each animal was kept for at least 2 days without handling and food deprivation to habituate to the novel situation of single-housing, cage enrichment and inverted dark-light cycle. After food restriction, mice underwent a strict handling protocol prior to experiments, performed for 1 week two times per day for at least 15 min, followed by habituation to the experimental setup in silence and darkness two times per day for four times (2 × 10, 1 × 15, 1 × 20 min) with pellets placed in the feeder bowl. Auditory training in the sound frequency discrimination task at a baseline frequency of 10 kHz was conducted two times per day with a minimum of 1.5 h in between, introduced with relatively short waiting times (0.2–0.7 s) and without sham trials. The waiting time was gradually increased until a stable performance (70% correct minimum) at waiting times between 1.25 and 5.25 s, which usually lasted 3–7 days (6–14 sessions), was observed in several consecutive sessions. A training session was terminated after 30 min or 40 received rewards. For more details on training procedure or frequency discrimination thresholds, see Rogalla et al. (2020).

##### Sound frequency discrimination task

Animals had to report a change in frequency within a continuous sequence of tone pips, presented with a rate of 5 Hz with a roving of levels between 60 and 66 dB SPL (randomly drawn) to avoid the detection of differences in loudness when the shift in frequency occurred. The shift was +0.241 octaves relative to the baseline frequency, which was between 10 and 18 kHz in steps of 2 kHz, presenting one frequency per session, randomly chosen. Multiple frequencies were presented to each mouse to ensure that animals would display flexibility when it comes to baseline and target frequency for a smooth transition to optogenetic activation. During training, a baseline frequency of 10 kHz was presented. Once the sound frequency discrimination task was learned, baseline frequencies could be exchanged without observing an impact on performance.

#### Optogenetic activation

Two 465 nm compact LED modules on a dual LED commutator (PlexBright, Plexon Inc.) were placed at the ceiling of the booth, approximately 1 m above the center of the circular runway. LEDs were independently connected to LED drivers (LEDD1B T-Cube, Thorlabs), on which the maximum current was set to 200 mA. Similar to sound cues, light pulses were generated via MATLAB and delivered using the sound card connected to the PC.

Light was delivered using optical patch cables (0.66 NA; PlexBright, high-performance fibers and LC ceramic ferrules, Plexon Inc.). Ceramic sleeves were used to connect cables and implants. The functionality of each cable was tested prior to each session. Stubs were gently connected without fixation of the implant or the head of the animal and covered with a black tube to additionally avoid visual detection of light pollution from the connection point.

##### Simple detection of optogenetic activation

To evaluate the usability of our paradigm and to test for the ability of 2-point detection, animals performed a simple detection task of a short pulse train in silence for one outlet (number of pulses: 4; pulse duration: 1 ms; rate: 13.8 Hz; light level: ∼6.5 mW). After achieving stable performance for at least two sessions, the light level was varied (∼0.6; 1; 2; 6.5 mW). If a significant detectability was still observable for the lowest light level, another combination of light levels was presented (∼0.09; 0.2; 1.5; 3.9; 5.2 mW) until reaching a subthreshold level (non-significant according to the Chi-squared statistics). Each light level was presented at least 20 times (two independent sessions). These experiments were then repeated for the respective other outlet. Which outlet was chosen for the first round was random. The whole detection period usually lasted 2–3 weeks.

##### 2-point discrimination task

The animals had to report a change of stimulation point within a continuous sequence of light pulses (number of pulses: 2; pulse duration: 0.1 ms; rate: 5 Hz). Usually, the ventral outlet represented the baseline point of activation, and each pulse (target and baseline) was presented with a level of ∼2.2 mW, if not stated otherwise.

If a stable performance could not be observed after three consecutive sessions for an equal light level in both points, the level of the baseline stimulation point was reduced (25, 50%). In a subset of experiments, random light level roving was introduced: light pulses of baseline and target were presented at different levels drawn from a uniform distribution of ±10% of the chosen center light level.

The period for these experiments strongly depended on the performance of each individual, lasting from 1 week (M4) to 4 months (M6).

##### Control task

To investigate if a visual cue leads to a significant performance in optogenetic experiments, a control task was implemented, partly adapted from Wrobel et al. (2018). Animals had to perform a simple detection task in five consecutive sessions with the highest light level from the psychometric experiment (∼6.5 mW), but with targets appearing with 50% probability in either one of the two outlets. In the second session, the light path of the dorsal outlet was blocked using black sponge rubber at the connector of stub and cable. During the third session, both outlets were enabled again, followed by the fourth session in which the other path (ventral outlet) was blocked with the same procedure. The fifth session was then conducted without blockage. During the blocked sessions, the light stimulus in the blocked point should trigger the same response as in the unblocked if the target is perceived as a visual cue from reflections at the implant or the chamber. This period lasted 2.5 days.

##### Control animals

To control for the necessity of the opsin, two animals have been trained, transfected and implanted as the other remaining animals, but were injected with a control construct, encoding only GFP (rAAV5-CAG-GFP). Experiments were conducted in the same order as for most of the experimental animals (M1–M5): sound frequency discrimination (including sessions where animals were attached to patch cables for habituation); followed by detection of optogenetic activation. In the latter, control animals were repetitively tested in six consecutive sessions. For the control animals, a click detection task was conducted within the next step to test whether these animals were able to switch between cues.

### Data analysis and statistics

In all experiments, for each session *i* and stimulus class *s*, the sensitivity *d*′ was calculated as:


d′i,s=z⁢(Hi,s)-z⁢(F⁢Ai),

where *z()* is the inverse of normal cumulative function, *H*_*i,s*_ is the hit rate *P(response*| *stimulus s)* for the stimuli with parameters *s* in the *i*-th session and *FA*_*i*_ is the false alarm rate *P(response*| *sham)*.

To discriminate between significant and non-significant detection, a Chi-square (χ^2^) statistic for the 2 × 2 contingency table (“go” and “no-go” responses during trials and sham trials) was calculated as:


$$x_{c}^{2}=\sum \frac{{\left(O_{i}-E_{i}\right)}^{2}}{E_{i}},$$

where *c* is the number of degrees of freedom, *O_i_* are the observed and *E_i_* the expected values for the *i*-th session.

### Model for the spread of neural activation

In order to estimate the overlap of neural activation for two stimulation sites, we conceived a 3D model for light spread and neural activation at each voxel. The light spread was modeled as an exponential decay of the light power density *p* along the fiber axis *z* (Al-Juboori et al., 2013):


$$p\,\left(z,\theta \right)=I_{0}\,e^{-\mu_{e\,f\,f}\,\left(\theta \right)\,z},$$

where *I*_0_ is the respective power density at the fiber tip and μ_*eff*_(θ) the effective decay constant at the angle θ. μ_*eff*_ was estimated separately for each angle θ using data from Gysbrechts et al. (2016). The resolution of the model was 10 × 10 × 10 μm per voxel, with corresponding θ and μ_*eff*_*I*. We defined neural activation of a voxel as the proportion of neurons within the voxel that fired as least one spike in response to the light stimulus. We used data for the light-dependence of photo currents and spike probability for ChR2 from Mattis et al. (2012) to estimate the activation *a* at each voxel, depending on the light power density *p*:


$$a\,\left(p\right)=\frac{1}{(1+e^{{-}^{*}\beta (\log\left(p\right)-p_{50}}},$$

with the slope parameter β = 0.2624 and 50% activation at the power density *p*_50_ = 0.814 mW/mm^2^. The activation *a* can be interpreted both as the probability of a neuron in a given voxel to be activated or as the number of neurons in the voxel responding to the stimulus. Using the voxel-resolved neural activation, we could estimate the number of “coactivated” neurons, defined as the number of neurons that would be active after stimulation from either of the two simulated outputs as the joint probability (or activation) of a neuron to fire at least one spike in response to both outlet 1 and 2: *a*_1_ × *a*_2_. By varying output light power and distance between light outlets we could simulate the relative amount of coactivated neurons by taking the sum over all voxels:


$$\frac{\sum_{z}\sum_{\theta }a_{1}\,\left(z,\theta \right)\,a_{2}\,\left(z,\theta \right)}{\sum_{z}\sum_{\theta }a_{1}\,\left(z,\theta \right)}.$$

## Results

### Freely moving mice detect optogenetic activation of the auditory midbrain

We devised a reward-based operant Go/No-Go paradigm to evaluate optogenetic activation at two different points along the ICc tonotopy of mice and compared the results with those of sound stimulation in the same animals. To verify the ability of each animal to perform a listening task using complex and continuous discrimination cues, mice were first trained in a sound-frequency discrimination task prior to implantation. After successfully performing (Chi-squared statistics) in the sound discrimination task with multiple baseline frequencies, mice were injected with a viral construct into the right ICc (rAAV5-CAG-ChR2-GFP) and implanted with two separated optical outlets, terminating at different depths, 700 μm apart (see Figures 1A–C). After recovery, testing in the sound-frequency discrimination task was repeated to account for habituation and comparable hearing ability post-surgery.

To test for detectability of optogenetic activation of the auditory midbrain, the detectability of a simple cue was measured at one of the two stimulation points in eight animals. The mice indicated the presence of a train of four light pulses (13.8 Hz stimulation rate, 465 nm wavelength, ∼6.5 mW power). Seven out of eight test animals reliably detected optogenetic activation (Chi-squared test), shown as the individual overall sensitivity (Figure 1E). One animal failed to switch from sound to light stimulation (M8, overall sensitivity *d*′ = −0.1375, three sessions, not displayed). However, in contrast to previous studies (Ceballo et al., 2019; Marshel et al., 2019), detection of optogenetic activation in the remaining seven animals did not require excessive training or habituation, and response to light stimulation was rapid (typically 1–2 sessions). These results demonstrate the suitability of our paradigm to evaluate artificial sensory stimuli.

### Optogenetic activation can be detected at two well-separated positions within the IC tonotopy

**FIGURE 2:**
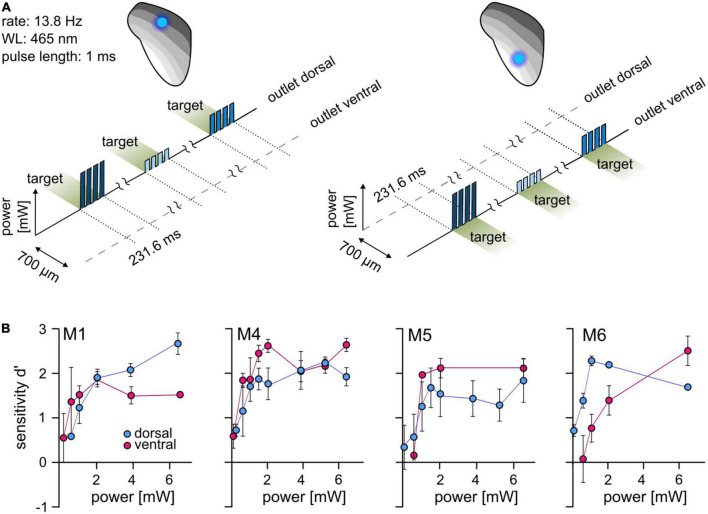
Optogenetic activation at two well-separated points along the IC tonotopy. **(A)** Psychometric paradigm. To investigate the detectability at two points of activation, animals performed a simple detection paradigm. The animals had to detect a light pulse train with a varying light level. Sessions were performed for both stimulation points independently. **(B)** Psychometric measurements. The mean *d*′ at different light levels (absolute power, *y*-axis, from ∼0.08 to ∼6.5 mW, absolute values) is shown for the dorsal (blue) and ventral (magenta) stimulation point. Error bars are ±SEM.

For differential stimulation and establishing complex artificial stimuli in the central circuits, optogenetic activation at more than one point within the tonotopy is required. We used a psychometric approach to test whether two points can be stimulated separately within the tonotopy of the ICc. Animals had to detect simple stimuli with a varying light level at each stimulation point separately (dorsal and ventral outlet, Figure 2A). In the four animals that completed these tests, optogenetic activation elicited excitation at both stimulation points and the sensitivity increased with increasing light level (Figure 2B). No systematic difference between outlet positions was observed, indicating that optogenetic activation can be detected at different points along the tonotopic axis of the IC.

### Mice discriminated between two points of optogenetic activation along the IC

**FIGURE 3:**
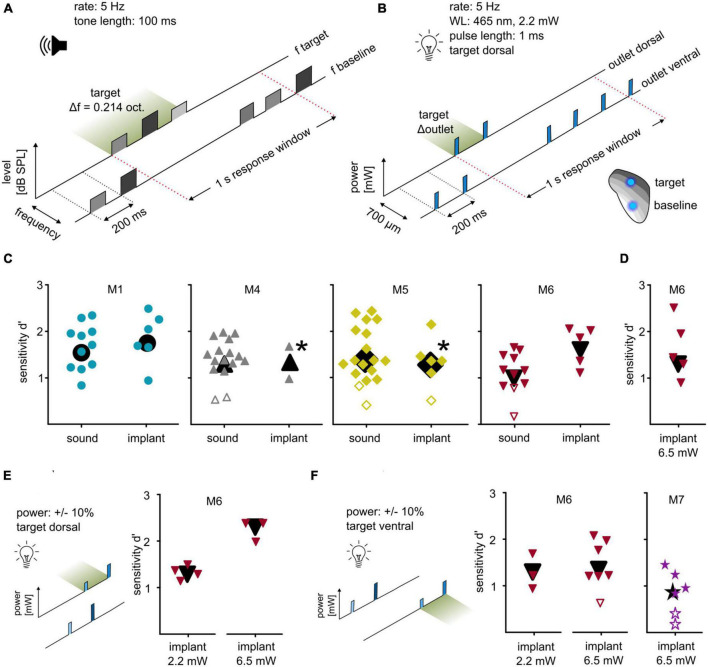
Differential optogenetic activation of the ICc. **(A)** Sound frequency discrimination. Animals had to indicate a change of frequency within a continuous sequence of tone pips (rate: 5 Hz; duration: 100 ms). One session comprised tones of a baseline frequency with a target frequency change of ±0.214 octaves. Tone levels were drawn from a uniform distribution between 60 and 66 dB SPL (level roving). **(B)** Two-point discrimination. Animals had to indicate a change in a continuous sequence of light pulses, achieved by the change of the stimulating outlet (465 nm, level: ∼2.2 mW; rate: 5 Hz; pulse duration: 1 ms; distance: 700 μm). **(C)** Behavioral performance. Sensitivity index *d*′ for all animals that were used in both paradigms is shown. The response toward post-surgery sound (left) and optogenetic activation (right) is plotted for each individual session, black symbols represent overall sensitivity. If animals did not succeed in discriminating the two stimulation points, the light level of the baseline outlet was reduced [M5: target power: 2.2 mW, baseline power 50% of target level; M4: target power: 2.2 mW, baseline power 25% of target power (upper point) and 50% (lower point)]. Note that M4 lost the implant afterward. Open symbols represent sessions with non-significant sensitivity. **(D)** Two-point discrimination at higher light levels. M6 additionally performed behavioral sessions with a power of ∼6.5 mW. **(E)** Two-point discrimination at roving light levels. For M6, the optogenetic paradigms were additionally applied using the same target position but with a roving of power (left: ∼2.2 mW; right: ∼6.5 mW; both ±10%). **(F)** Two-point discrimination with exchanged stimulation points at roving light levels. These paradigms were repeated but with exchanged target and baseline position (left). Same was applied for animal M7 (right) but at a high power only (∼6.5 mW ±10%).

Next, we investigated whether optogenetic activation at two points can be used to introduce more complex stimuli by evaluating discrimination between these two points, comparing the results with the post-surgery sound-frequency discrimination task. In the auditory task, the animals indicated a change of frequency within a continuous sequence of tone pips (Figure 3A). Random roving of tone levels between 60 and 66 dB SPL was applied to avoid potential level cues. This task was later mimicked by optogenetic stimulation: animals indicated a change of stimulation point (outlet position) within a continuous sequence of blue light pulses (∼2.2 mW). Stimulation at the dorsal outlet represented the target, whereas stimulation at the ventral outlet served as a baseline (2-point discrimination task, Figure 3B). M1 and M6 immediately performed well in this 2-point task with significant sensitivity (Figure 3C), whereas for M4 and M5 baseline stimulation needed to be adjusted for the mice to successfully discriminate (M5: 50% of background, *d*′ = 1.36; M6: 25%, *d*′ = 1.98). All background and target light levels were well within mouse detectability (Figure 2B). All animals that were used in these particular experiments displayed a similar sensitivity under both conditions.

The light level for the 2-point discrimination task (∼2.2 mW) was chosen as a clearly detectable level (see Figure 2B), but low enough to avoid exciting larger tonotopic areas. To test discriminability dependence on the power, one animal was tested at a level of ∼6.5 mW and successfully discriminated between the two stimulation points (M6, Figure 3D), indicating that discriminability did not strongly depend on overall irradiance.

### Discrimination of two activation points within the IC tonotopy can be performed with a roving light level and works in both directions

The discriminability demonstrated here may derive from differences in light delivery (LED/patch cable/fiber stub), from a difference in opsin expression, or from a combination of the two. To test this, light level roving was introduced, like the level roving in the sound frequency discrimination task. For two animals (M6 and M7), light pulses of baseline and target were presented at different levels drawn from a uniform distribution of ±10%. For one animal, low and high light levels were tested separately (M6, Figure 3E). M6 significantly detected the change in stimulation point immediately in both tasks without any additional training or habituation.

The discriminability may also depend on chosen baseline/target position. To test whether baseline and target points can be exchanged, the same condition (roving of light level) was applied but with switched target and baseline positions: ventral as target, dorsal as baseline (Figure 3F). M6 discriminated between these for both low (2.2 mW ±10%) and high (6.5 mW ±10%) light levels. M7 only performed well with the high light level. Although M7 performed poorer than M6, its overall sensitivity was significant (Figure 3F). Thus, discriminability of optogenetic IC activation can be performed using both positions as targets and with a roving light level.

### Optogenetic activation is not detected visually

**FIGURE 4:**
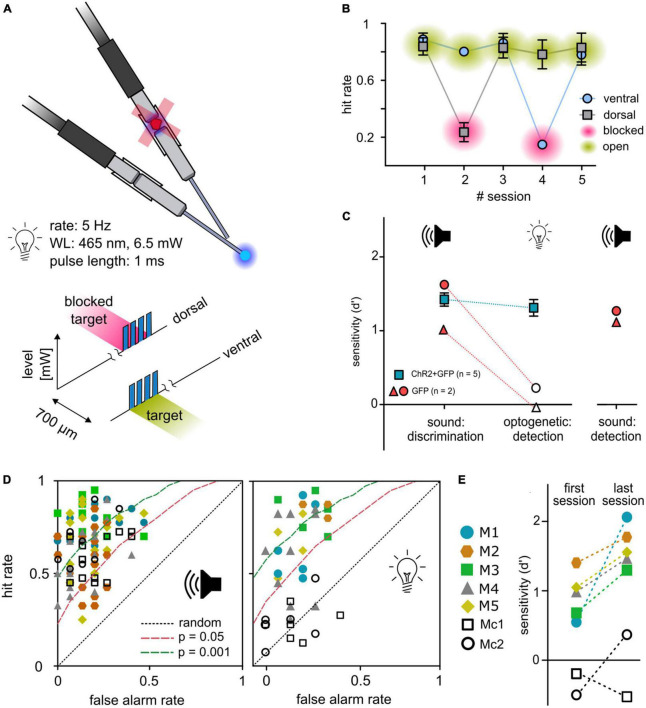
Control experiments. **(A)** Control paradigm. To control for a possible visual light detection, animals performed a detection paradigm with a constant light level (6.5 mW) in which they had to detect a short pulse train as target in either one of the two outlets. In each second session, one outlet was blocked (magenta) at the connector from patch cable to animals implant. **(B)** Behavioral performance. Mean hit rates (*y*-axis) are shown for both outlets and five consecutive sessions (*x*-axis) for animal M1/5/6. Error bars are ±SEM. **(C)** Control animals. Two animals (gray circles) were injected with a sham virus without the construct for ChR2 (rAAV5-CAG-GFP). Their behavioral performance (sensitivity *d*′, *y*-axis) is shown in comparison to the experimental group (blue squares, mean ± SEM) for sound frequency discrimination (post-surgery) as well as for the light detection paradigm. To evaluate if the reduced performance of control animals in the detection paradigm for optogenetic activation is caused by reduced generalization, control animals additionally performed a click detection paradigm afterward. **(D)** Receiver operating characteristic: sound vs. activation. Open symbols depict control animals; filled symbols depict animals from the experimental group. Distribution of all sessions according to the false alarm rate (*y*-axis) and hit rate (*x*-axis) for sound frequency discrimination (left panel) and detection of optogenetic activation (right panel). Dashed lines represent levels of significance according to the Chi-squared statistic. **(E)** Progress in performance over all sessions. Open symbols depict control animals; filled symbols depict animals from the experimental group. Performance for detection of activation is shown as sensitivity of the first vs. the last session.

To avoid possible visual detection of light stimulation, a ceiling blue-light LED strip continuously pulsed at the stimulus rate during each session. Although this light should mask visually detectable blue light pulses, the possible detection of visual cues from fibers or connectors remains. Thus, in all animals that completed the 2-point discrimination, a control task of a set of five independent sessions to detect a light-pulse train in either one of the two outlets with 50/50% probability was conducted (see Figure 1E). In every second session, black foam blocked the implant’s connector (Figure 4A). For open outlets (green), mean hit rates remained relatively stable independent of outlet position (dorsal or ventral). In contrast, the hit rates dropped to chance level for both outlets when blocked (magenta) but reemerged during the following session (Figure 4B), indicating that the light stimulation was not visually detectable.

### Detection of optogenetic activation requires the expression of ChR2

Independent of light-sensitive ion channels, thermal effects of light delivery might elicit a neuronal response and enable the detectability of light stimulation (Owen et al., 2019). To control for the necessity of the opsin, 2 animals were trained, transfected, and implanted as previously described, but were injected with a control construct (rAAV-CAG-GFP, Figure 4C; cM1, cM2). Both animals significantly detected a change in sound frequency given as overall sensitivity (Figures 4C, D, left panel). During the light-detection task (compare Figure 1E), experimental animals detected optogenetic activation, whereas stimulation failed to elicit a significant response in control animals (Figures 4C, D, right panel and Figure 4E). To prove that this was not based on reduced generalization but on the absence of an observable target, light pulses were exchanged with broadband clicks, using the same temporal properties as the optogenetic detection stimulus. Control animals immediately detected click stimuli in the first session and showed significant overall sensitivity for six consecutive sessions (overall *d*′, Figure 4C), indicating that the insensitivity of the control animals was not based on a lack of generalization.

## Discussion

In this study, our implant differentially stimulated two points along the ICc tonotopy and permitted to generate behavioral responses to complex artificial cues using optogenetic activation. We tested the ability of mice previously trained in a sound discrimination task to detect two independent points of optogenetic activation within the ICc, and to discriminate between them. First, we performed a general detection task of optogenetic activation in the ICc to evaluate the suitability of our paradigm. Seven of eight animals detected optogenetic activation in the ICc, demonstrating the suitability of this approach (Figure 1E). Second, we investigated the behavioral sensitivity (*d*′) toward optogenetic activation resulting from irradiance at two points within the ICc tonotopy (Figure 2). As shown for simple optogenetic activation of rodent spiral ganglion neurons (Wrobel et al., 2018) and the ICc (Guo et al., 2015), behavioral sensitivity in our animals increased with increasing irradiance, but at both stimulation points separately. Thus, optogenetic activation can differentially stimulate the subcortical central auditory pathway. Third, we demonstrated discrimination between points in a continuous activation task (Figure 3). To our knowledge, this study is the first systematic, long-term behavioral analysis of optogenetic activation in the auditory pathway and the first proof-of-principle for differential and behaviorally relevant optogenetic activation of the sub-cortical auditory pathway in freely moving, behaving animals.

### Variability of behavior as a consequence of failed generalization

In total, eight animals were injected with ChR2-AAV and implanted with two independent outlets along the tonotopy of the ICc. One animal did not generalize from sound stimulation to optogenetic activation (overall sensitivity *d*′ = −0.1375, three sessions, mouse M8), and was additionally tested in the click-detection paradigm, a stimulus that resembles the temporal pattern of the light stimulus (data not shown) and again failed to respond. Therefore, as the implant was placed correctly, the failure in the optogenetic task was likely based on poor behavioral flexibility (discrimination vs. detection). The remaining two animals, which successfully performed the optogenetic detection task but not the task involving differential stimulation, either had lost the implant during experiments (M3) or developed a health problem and experiments were terminated (M2, for more details see Supplementary Table 1).

Despite the fact that differential optogenetic activation of the ICc could be demonstrated here, we observed considerable variability between individual mice. For two animals, baseline light levels for the discrimination task had to be lowered, which indicates that these mice might have required additional level cues to discriminate between the two points of optogenetic activation. Since all mice were able to discriminate between tone pips of different frequencies, the limiting factor might not have been the discriminability of the optogenetic stimuli *per se*, but the variability in the efficiency of optogenetic activation, possibly due to differences in expression or exact placement of fiber terminals within the tissue. Further research will be required to make judgments about the actual percept elicited by optogenetic activation of tonotopic regions.

### Perception of optogenetic activation

Although animals that reached the final experimental stage discriminated two points of optogenetic activation, our study does not elucidate the exact percept. Two major factors of activation could have contributed to the discriminability: “level” and “(sound) frequency” cues. Level cues could depend on a change in the overall activation, while frequency cues would be based on a shift of excitation along the tonotopy.

If discriminability was based on level cues only, 2-point stimulation could have created a difference in the overall “loudness” of baseline and target, independent of spectral aspects. In that case, one needs to assume that stimulation at the two points resulted in widespread excitation in the entire ICc and in a broad spectral percept and that the discrimination is purely performed by comparing the resulting “loudness” level between outlet a and b. Indeed, with the currently chosen approach, we cannot rule out that purely loudness level cues drove the observed discriminability in our experiments. Perceived loudness of artificial auditory cues is difficult to measure (especially in mice) and would require knowledge about central neuronal correlates of loudness, especially when these are generated at the IC level. Even under electrical stimulation, level cues could be derived and further research will be required to establish a proxy for “loudness” level cues in central auditory implants. However, by using light levels in the 2-point discrimination task that were clearly within the detectable range for both outlets (Figure 2), we can rule out that gap detection or simple detection performance (meaning that presenting a light stimulus at one of the two outlets did not result in any neuronal activation) led to the observed performance.

An alternative consequence of 2-point activation of different levels for baseline and target could have been a widening or narrowing of the activation area that altered the spectral bandwidth of the percept when switching from baseline to target and vice versa. It is less probable that exclusively spectral differences derived from the anatomical position of the activation points within the IC tonotopy caused the discriminability of target and baseline. Here, discriminability between two auditory percepts differing only spectrally would have been possible without the adjustment of the baseline irradiance in animals M4 and M5.

Although the baseline amplitude had to be reduced for two animals to observe a reliable sensitivity, two other animals even performed the task with a roving of baseline amplitude and thereby a roving of irradiance for each single light pulse, disproving discrimination based on alterations in the overall irradiance for these animals. Thus, different percepts elicited in the different animals, accompanied by the animals’ ability to generalize, may also explain the observed variability. Nevertheless, all these scenarios constitute considerably more complex stimuli than simple activation at a single position, as used in previous studies.

In summary, it cannot be fully determined how both spectral and loudness cues contributed to the discriminability. Further physiological experiments revealing the responses of ICc neurons to optogenetic activation and sound will allow more detailed assumptions concerning the artificially generated percept.

### Suitability of the paradigm for the long-term evaluation of optogenetic activation and stability of performance

**FIGURE 5:**
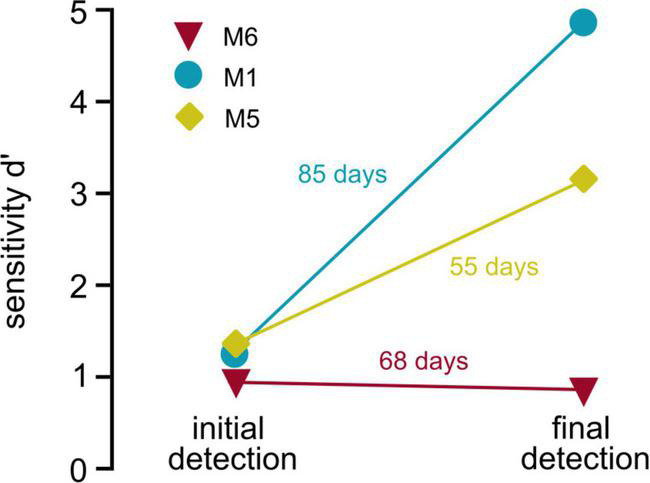
Stability of behavioral performance. Behavioral performance over time: Sensitivity (*d*′, *y*-axis) is shown for initial detection of optogenetic activation (left, overall sensitivity over multiple sessions, data obtained from Figure 1E) vs. the sensitivity during the last behavioral session (last control session, obtained from Figure 4B) of three animals is shown. Numbers next to the lines depict number of days the implant was carried between both sessions.

Previous studies of optogenetic activation of the auditory pathway in freely moving mice used the shuttle box paradigm (Guo et al., 2015; Wrobel et al., 2018). Although the avoidance-learning procedure provides significant advantages, these paradigms cannot be easily applied to all research questions. In experiments using complex schedules requiring repetitive testing of animals, reward association positively influences behavioral outcomes (Patterson-Kane et al., 2008), and the reduction of stress and avoidance cues improves behavioral performance in mice (Havenith et al., 2019). In our paradigm, animals were not restrained during the process of implant-cable attachment to reduce forces on implants and to avoid negative associations with handling. When comparing behavioral performance in the detection task for three animals that performed both in the initial and the control task (depicting beginning and end of optogenetic schedule, Figure 5), it is shown that behavioral performance either remains relatively stable (M6) or drastically increases over time (M1 and M5), even with almost 3 months in between experiments (M1). Repetitive handling, however, made the paradigm time consuming, which should be considered when applying our approach. Nevertheless, the different evaluations covered by our study demonstrate the flexibility and advantage of the paradigm for the long-term evaluation of optogenetic activation in freely moving animals.

### Future optimization of optogenetic activation of the auditory midbrain

The ICc acts as the midbrain hub for the integration of monaural and binaural ascending pathways, ascending projections to higher auditory and non-auditory areas, and, additionally, exhibits an intercollicular pathway (Syka et al., 2000; Malmierca, 2004; Gruters and Groh, 2012). Within its circuitry, approximately 25% of all neurons are GABAergic; the remaining are glutamatergic (Merchán et al., 2005; Ito et al., 2011; Beebe et al., 2016; Naumov et al., 2019). Thus, targeting ChR2 expressed in the IC under the control of the pancellular promoter CAG will excite both excitatory and inhibitory neurons. With regard to the potential use of optogenetics in auditory prostheses, cell-type selectivity could stimulate excitatory neuronal populations in different iso-frequency laminae without altering the entire network dynamics, thus evading a problem of electrical stimulation of the IC (Quass et al., 2018). Although our approach did not specifically target excitatory neurons, generalization between sound- and optogenetic activation was rapid, and sensitivity values did not differ greatly. That the activation of mixed neuronal populations did still lead to significant discriminability in our approach might be due to the relatively large distance between activation points along the tonotopy (∼700 μm). Greater excitatory specificity might be required for higher spatial resolution when using stimulation at smaller distances.

**FIGURE 6:**
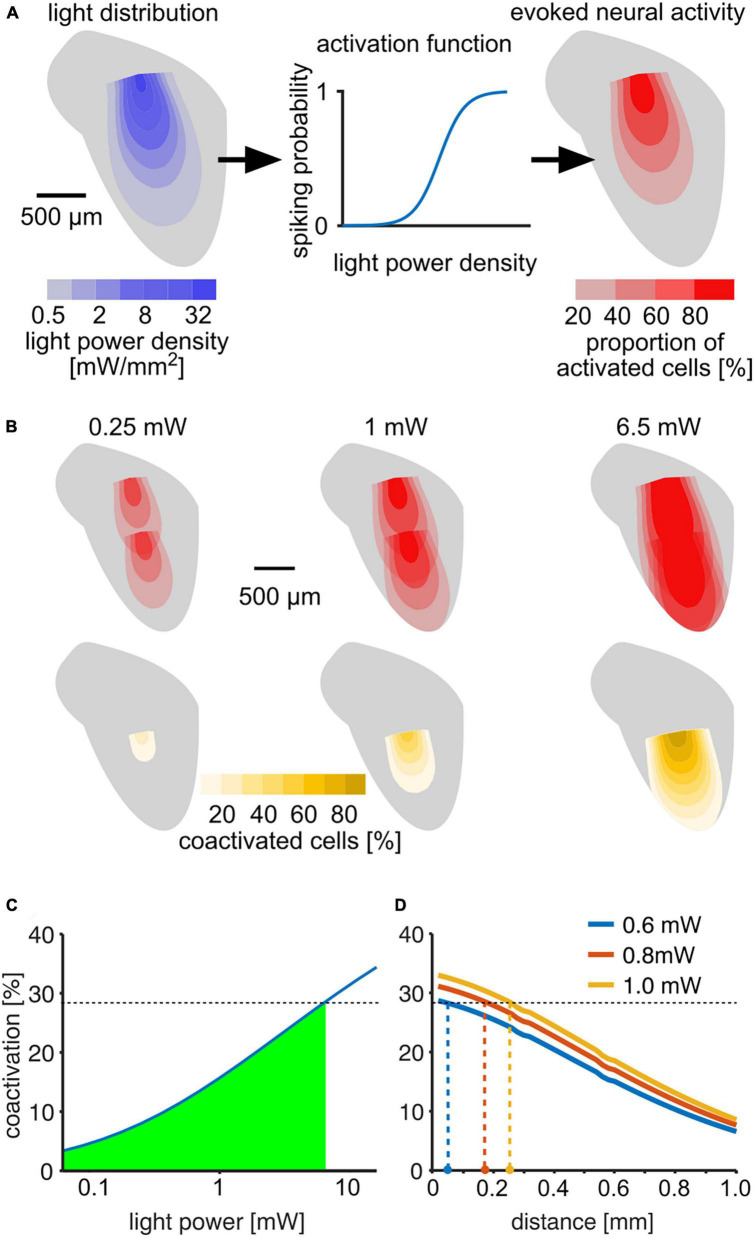
Estimation of light distribution and spread of neural activity throughout the ICc. **(A)** Model layout: light distribution was modeled using an angle-dependent exponential decay of light power density (Mattis et al., 2012; Gysbrechts et al., 2016). Left: color-coded 2D profile of the light distribution cone estimated for a 1 mW stimulus. The estimated light power density was used to estimate the probability to fire at least one spike for neurons in each voxel (middle). The activation function was fit using data from Mattis et al. (2012). Right: 2D profile of activation cone for a 1 mW stimulus. **(B)** Upper row: neural activation profiles at three different output light levels for consecutive stimulation at two different outlets within the IC. The distance of 700 μm corresponds to the setup used in our experiments. Color scale is the same as in panel **(A)**. **(B)** Lower row: profiles of percentage of neurons activated by both stimuli (“coactivated”) in each voxel. **(C)** Percentage of neurons that are activated by both outlets compared to the overall activation by a single outlet as a function of light power. The green area marks coactivation that is smaller than at 6.5 mW, for which animals were able to discriminate between stimulation at the two outputs (Figure 4). **(D)** Estimation of minimal distance of the light outlets for lower light levels, based on the assumption that % coactivation limits the discrimination and that mice successfully discriminated the 6.5 mW stimulus with 28.5% coactivation. Colored dots at the *x*-axis are estimates of discriminable outlet distances at light levels at and above the detection thresholds.

To estimate an upper bound on how the stimulation activates different areas of the IC, we developed a computational model of light activation (Figure 6A). Based on estimates of light spread (Gysbrechts et al., 2016) and neuronal activation threshold (Mattis et al., 2012), we calculated the percentages of neurons activated at different distances from the fiber tips (Figure 6B). Up to 29% of all optogenetically excited neurons were activated by both outlets at 6.5 mW, for which we observed successful discrimination (Figure 6C). Accordingly, mice can tolerate as least this level of overlap, and we accepted 29% as a conservative estimate for the upper bound for discrimination. Our detection experiments revealed that mice detected much lower light levels (Figure 2), and co-activation strongly depended on light level. Thus, we expect that light outlets can be moved considerably closer while preserving discriminability (Figure 6D). At 0.6 mW, for which four out of eight stimulations resulted in behavioral detection sensitivities *d*′ = 1 in our experiments, stimulation at outlets spaced as close as 50 μm should be possible. But even at 1 mW, which was clearly above the detection threshold for seven out of eight tested outlets, a distance of 250 μm should be resolvable. Based on previously published data on frequency mapping in the mouse IC (Stiebler and Ehret, 1985; Portfors et al., 2011), we expect a difference in center frequency (CF) of approximately 0.3–0.8 octaves between the two fiber tips along the implantation axis (700 μm apart). Accordingly, 100 μm distance would correspond to a 0.04–0.11 octave difference in CF that can be discriminated. If implanted in a human ICc, the distance we used in mice (700 μm) would correspond to a resolution along the tonotopic axis of ∼0.35 octaves (Ress and Chandrasekaran, 2013). Thus 100 μm would correspond to ∼0.05 octaves (or 3.5%) in the human IC, even without expression limited to excitatory cells. However, further experiments are necessary to test discriminability and excitatory specificity.

Another path for improvement is to avoid the relatively slow kinetics of ChR2. ChR2 recovery requires 10–20 ms (dependent on stimulation rate and physiological conditions; Grossman et al., 2010; Schneider et al., 2015). Regarding the potential of optogenetics for restoring auditory function, ChR2 might not be a suitable ion channel for precisely timed processing; other channels such as Chronos should achieve more precise coding of auditory information (Guo et al., 2015; Keppeler et al., 2018). However, while the use of Chronos produced more synchronized neuronal responses in the mouse IC at rapid stimulation rates, it did not improve behavioral detectability compared to ChR2 (Guo et al., 2015). It remains to be seen whether ultra-fast ion channels can improve sensitivity for more complex optogenetic activation cues.

To furthermore achieve highest levels of spectral resolution, modifying light delivery could greatly improve stimulation outcome. Light delivery which considers the shape and orientation of isofrequency lamina could improve activation and reduce overlap. Tapered optical fibers for example, which not only reduce tissue damage but also enable dynamic modulation of direction and position of emission (for review see Fernandez-Ruiz et al., 2022), could be utilized to improve spatial resolution in optogenetic auditory implants.

### Perspective

The present study demonstrates that differential stimulation of the central auditory system is possible using optogenetics for the generation of complex, continuous sensory patterns. Further research is needed to reveal the actual network activity resulting from differential optogenetic activation of the sub-cortical auditory pathway. Behaviorally, it remains to be shown how the discriminability of two neuronal ensembles depends on the distance of activation outlets and the limits of spatial resolutions.

Our paradigm could be very helpful for future work on optogenetic restoration of sensory function since it provides a high level of flexibility and a reliable behavioral read-out of optogenetic activation over periods of several months. Our results demonstrate that optogenetic activation of sub-cortical sensory nuclei could be a powerful tool to restore sensory function in the auditory system and beyond.

## Data availability statement

The raw data supporting the conclusions of this article will be made available by the authors, without undue reservation.

## Ethics statement

The animal study was approved by the State Office for Consumer Protection and Food Safety/LAVES, license no. 33.19-42502-04-18/2802. This study was conducted in accordance with the local legislation and institutional requirements.

## Author contributions

MR and KH: conception and design of the work. MR, KH, and JS: critical revision. MR, JS, and AS: data collection. MR: data analysis, interpretation, and drafting the manuscript. KH: model design and analysis. All authors contributed to the article and approved the submitted version.

## Footnotes

1
https://github.com/Spunc/PsychDetect
