# Supplementary material for: Differential optogenetic activation of the auditory midbrain in freely moving behaving mice
Source: Front Syst Neurosci. 2023 Aug 31;17:1222176. doi: 10.3389/fnsys.2023.1222176 (PMC10501139; doi:10.3389/fnsys.2023.1222176)
Supplement: Supplementary file 1 [file Table_1.pdf]

table S1: conducted experimenst per animal

animal	experiments conducted					reason for termination
M7	sound	detection	discrimination			pilot animal of this study
M6	sound	detection	detection-2 points	discrimination	control	experiments completed
M1	sound	detection	detection-2 points	discrimination	control	experiments completed
M5	sound	detection	detection-2 points	discrimination	control	experiments completed
M4	sound	detection	detection-2 points	discrimination		lost implant during discrimination
M2	sound	detection				health issue
M3	sound	detection				lost implant during detection at first outlet
M8	sound					failed to respond in detection experiments

Mc1	sound	detection light	detection sound	experiment completed
Mc2	sound	detection light	detection sound	experiment completed

The table shows the conducted experiments per animal used in the study. The last column gives the reason of experimental termination for each mouse. Upper panel shows the schedule of test animals, whereas the lower panel shows the experimental schedule of control mice. "Sound" refers to the frequency discrimination training and experiments.
